# Thyroid cancer surgery during the coronavirus disease 2019 pandemic: perioperative management and oncological and anatomical considerations

**DOI:** 10.2217/fon-2021-0585

**Published:** 2021-08-25

**Authors:** Eleftherios Spartalis, Nikolaos Plakopitis, Maria Anna Theodori, Sotirios P Karagiannis, Dimitrios I Athanasiadis, Michael Spartalis, Georgios Boutzios, Stavroula A Paschou, Nikolaos Nikiteas, Theodore Troupis

**Affiliations:** ^1^Laboratory of Experimental Surgery & Surgical Research ‘N S Christeas’, Medical School, National & Kapodistrian University of Athens, Athens, 11527, Greece; ^2^Department of Anatomy, Medical School, National & Kapodistrian University of Athens, Athens, 11527, Greece; ^3^Department of Surgery, Indiana University School of Medicine, Indianapolis, IN 46202, USA; ^4^Department of Pathophysiology, Endocrine Unit, Medical School, National & Kapodistrian University of Athens, Athens, 11527, Greece; ^5^Department of Clinical Therapeutics, Alexandra Hospital, Medical School, National & Kapodistrian University of Athens, Athens, 11528, Greece; ^6^Second Department of Propaedeutic Surgery, Medical School, National & Kapodistrian University of Athens, Athens, 11527, Greece

**Keywords:** COVID-19, goiter, SARS-CoV-2, surgery, thyroid cancer, thyroidectomy

## Abstract

The coronavirus disease 2019 (COVID-19) pandemic has changed many aspects of our everyday lives and medical practice, including oncology treatment; thyroid cancer surgery is not an exception. The reported number of fine-needle aspirations performed during the first semester of 2020 was significantly reduced. Poorly differentiated, medullary and anaplastic thyroid tumors are considered important indications for immediate surgical intervention. By contrast, most well-differentiated carcinomas present slow growth, and thus surgery can be deferred for a short period of time during which patients are under active surveillance. Thyroid surgeries have decreased during the COVID-19 pandemic. Furthermore, prior to any intervention, negative COVID-19 status – with the use of a nasopharyngeal swab and reverse transcription PCR assay as the gold standard and chest CT scan as a complementary modality in some cases – must be confirmed to achieve a COVID-free pathway. Thorough preoperative assessment regarding both oncological and anatomical aspects should be performed to identify optimal timing for safe management.

Coronavirus disease 2019 (COVID-19), which is caused by SARS coronavirus 2 (SARS-CoV-2), was first reported in Wuhan, China, in December 2019 and was declared a pandemic by WHO on 11 March 2020 [1,2]. As of 5 April 2021, there have been 132,045,493 confirmed cases, of which 2,868,050 have been fatal [3].

To contain the rapid spread of the infection, governments around the globe have implemented social distancing, lockdowns, quarantines, travel restrictions and hygiene measures [4,5]. In addition to everyday life, psychosocial behavior and the economy, medical practice has, expectedly, also faced the implications of this pandemic [2,6]. Reallocation of resources to treat COVID-19 patients, leading to hospital bed and staff shortages, and prioritizing cases to reduce transmission and relieve healthcare systems have altered the standard of care for many illnesses [7–13].

The American College of Surgeons first recommended to postpone or cancel scheduled elective procedures [7,14,15]. Later, the American College of Surgeons issued guidelines for triage and management of oncology surgeries [7,11]. Despite the undoubtable benefits of oncology treatment, cancer patients are more likely to contract COVID-19 and are more vulnerable to complications [7,11,14]. Thyroid cancer patients are generally not an exception, as far as respiratory infections are concerned, demonstrating various mechanisms of cellular and humoral immunity suppression even in the case of differentiated thyroid cancer, although research suggests that these latter patients are no more susceptible to severe disease than the general population [16–18] Although some cancers require urgent operations, others do not, even though patients may understandably be afraid [4,10,11]. Thyroid tumors generally grow slowly [4,14,19]; in fact, observation of microcarcinomas is encouraged by the American Thyroid Association [4,20]. The aim of this study was to review thyroid cancer surgery framework shifts throughout the pandemic globally as well as the actual impact on clinical reality, diagnosis, treatment and follow-up.

## Methods

Eligible articles were identified by a search of the Medline (PubMed) electronic database and Cochrane Library up to 5 April 2021. The search strategy included the following keywords and MeSH terms: thyroidectomy or ‘thyroid surgery' or 'endocrine surgery' and cancer or oncology and covid-19 or SARS-CoV-2 or pandemic.

Abstracts of potentially relevant titles were assessed for eligibility, and full-length articles were selected for closer screening. The selection was limited to guidelines, recommendations and both prospective and retrospective studies describing original data written in English. Out of 38 results, 14 were considered to meet the inclusion criteria [1,4,7–9,18,21–28].

## Results

### Effects on diagnosis

Before approaching the effects of COVID-19 on surgery of thyroid gland cancers, it is essential to examine whether the diagnostic approach has changed during the pandemic. Zhang *et al.* found that during the period between January and February 2020 (pandemic phase I), when the alert level was at its highest in Changchun, China, fine-needle aspirations (FNAs) were conducted 99.7% less often than FNAs during the period between January and February 2019, and none of the tumors under examination were found to be malignant [21]. This gap was progressively shortened from the period between February and March 2020 (pandemic phase II) to the period between March and April 2020 (pandemic phase III). During phase III, FNAs were performed 30.1% less often than FNAs during the period between March and April 2019, and approximately half of the samples were malignant. Outpatient visits also showed a progressively smaller decline (from 93.3 to 18.3%) during those months.

Rana *et al.* compared all diagnostic cytology examinations performed from March to May 2020 – the time frame of national lockdown in India – with those performed during the same interval in 2019 and found a significant decrease in thyroid cytology samples, with no FNAs of thyroid tissue performed during this period [22]. It is clear that during the pandemic the number of FNAs significantly declined [21,22]. Nonetheless, it is of great importance to determine whether this reduction could potentially be harmful for patients.

Vrachimis *et al.* concluded that diagnostic procedures (FNA, ultrasound, scintigraphy) in patients with thyroid nodules can be postponed safely and established that a delay of a few months in diagnosis does not affect the outcome of thyroid cancer in a negative way [18]. However, the clinical picture should always be taken into account, and FNA should not be delayed in patients with a history or suspicion of medullary, anaplastic or metastatic thyroid carcinoma and significant symptoms of pressure to critical structures, dysphagia, dyspnea or dysphonia. The researchers also suggested teleconsultation as a way of informing patients about the reasons leading to alteration of the procedure and clarifying that every decision is made based on a thorough assessment of risks and benefits.

### Recommendations for surgical triage

After the outbreak of the COVID-19 pandemic, and because of the high risk of infection in patients and surgeons, concern arose regarding the proper selection of cancer patients for surgical management. To overcome these difficulties, some medical groups have proposed parameters that contribute to prioritizing surgical cases [1,4,7,8,18,23,24]. Among the suggested parameters, histological and clinical characteristics are those commonly utilized. First and foremost, acute airway compression is an indication for urgent surgery, which should be carried out even in acute phases of the pandemic, when the burden on hospitals is greatest [7,18]. With regard to histology, anaplastic, poorly differentiated and medullary thyroid cancers are those that most often require urgent surgical intervention [1,4,7,8,18,23,24]. In addition, patients with locally aggressive tumors with adverse features, such as invasion of surrounding vital structures (e.g., recurrent laryngeal nerve, trachea, esophagus, major vascular structures) and large, compressive or fast-growing differentiated tumors with concurrent nodal involvement, should be considered candidates for surgery. By contrast, surgical management for most well-differentiated carcinomas, which are generally slow-growing, can be safely postponed for approximately 3–6 months. During this period, patients can remain under active surveillance and be evaluated at predefined time intervals. Should surgery be impeded, alternative therapies, including chemotherapy, targeted therapies and ^131^I, mainly for the treatment of anaplastic or poorly differentiated carcinomas, can occasionally be suggested [1,4,7,18 23]. However, these therapeutic choices render thyroid cancer patients susceptible to COVID-19 infection and more severe illness [18]. Therefore, there is conflict regarding whether these modalities should be considered part of the therapeutic algorithm. Although there are those who recommend these therapies even as first-line therapy, others are opposed to using them unless offered as palliative care [23,24]. However, as surgeries decrease – including thyroid cancer surgeries – as part of the healthcare regulations put in place after the onset of the pandemic, the aforementioned modalities can, circumstantially, benefit high-risk patients unable to undergo surgery. Thus, despite the threat of COVID-19 infection, they should be offered in selected cases after performing an individual risk–benefit analysis [18].

Another parameter examined by Jozaghi *et al.* are the phases of care published by the American College of Surgeons [7]. Specifically, management of surgical cases is dependent on regional prevalence and is aligned with the hospital's capacity to perform selected surgeries based on intensive care unit capacity and available supplies. Finally, other researchers have defined scheduling levels, each corresponding to an ideal surgery time and urgency level (surgical tier class) correlated with the prognosis, to select patients [1,23].

### Preoperative assessment

Before proceeding with any invasive therapeutic maneuver or surgical intervention, the COVID-19 status of the patient should be evaluated for the safety of both patient and personnel [1,4,8,9,21,24,25]. A nasopharyngeal swab sample and subsequent reverse transcription PCR assay are the gold standard assessment modality [1,8,21,24,25]. Should the patient test negative while there is still clinical suspicion, the test is repeated within a 24- to 48-h interval [1]. Because of limited diagnostic value of the reverse transcription PCR method, conduction of a complementary chest CT has been recommended by some studies [1,21]. If the patient tests positive, the surgery is postponed until resolution of symptoms and normalization of laboratory test values [21,24]. In addition, clinical evaluation [1,8,24] and blood and biochemical testing are suggested by some researchers not only for COVID-19 status screening but also for a general preoperative assessment [1,25]. Preoperative vocal cord examination should also be performed with the appropriate personal protective equipment [1].

### Impact on surgical services

Some medical groups have statistically analyzed the actual effect of the pandemic on thyroid cancer surgery. Specifically, Lombardi *et al.* performed planned surgeries on 14 patients over a period of 4 weeks without delay, accounting for one-third of the usual surgical workload, and nonurgent cases (n = 79) were postponed [8]. Bakkar *et al.* reported 12 patients who successfully underwent surgery as scheduled [9]. In addition, according to de Pelsemaeker *et al.*, oncology procedures, including those for thyroid cancer, were classified as urgent and were allowed to continue [26]. Nevertheless, a statistically significant decrease in thyroidectomies was noted in a comparable time frame. In the retrospective analysis by Zhang *et al.*, results are presented based on the three public health emergency phases of the pandemic [21]. In the first phase, no surgeries were performed, and in Phases II and III, a significant decrease in both inpatients and malignant thyroidectomies was noted and mean surgical time was decreased in comparison with the same period in 2019 [8]. Moreover, in the cohort study by Wai *et al.*, a reduction in thyroid procedures was reported, with an increase in operative time [27].

### Impact on follow-up

No perioperative COVID-19 transmissions were reported [8,21,27]. Wai *et al.* [27] and Lombardi *et al.* [8] came to the conclusion that there was no significant difference in the number of patients experiencing postoperative complications during the pandemic. By contrast, Zhang *et al.* observed that vocal cord paralysis significantly increased in Phase III [21].

Aygun *et al.* recommend dealing with patients at risk of postoperative hypocalcemia on an outpatient basis and treating patients with severe hypocalcemia in the hospital until it can be determined that discharge carries only a minimal readmission risk [1]. It is understood that thyroid function tests may be difficult to carry out because of the global pandemic [23]; hence, Aygun *et al.* recommend treating patients with levothyroxine until further testing becomes available [1].

Zhang *et al.* found that the mean postoperative hospital stay was significantly reduced by 0.4 days compared with the same time frame in 2019 [21]. With regard to longer-term follow-up, Baud *et al.* recommend teleconsultation as a proper way to ensure continuity of care while limiting the risk of SARS coronavirus 2 transmission [23]. Additionally, the researchers advise that follow-up blood and imaging tests be performed by primary care providers.

## Discussion

### Protocol variations

The pandemic has affected many aspects of medical practice, including diagnostic procedures, surgeries and follow-up protocols [1,4,7–9,18,21–28]. This review found that the number of surgeries and FNAs was generally decreased. However, there was a discrepancy in the degree to which these numbers declined between the different articles used for the purposes of this review. This finding is probably a reflection of the various effects of lockdown restrictions applied in different countries at different stages of the pandemic. The differences between the articles may also be attributed to recommendations that were rushed to publication early in the pandemic and based on anecdotal previous experiences with coronaviruses, pandemics and natural disasters [8,9,15,21,22,25–27,29]. However, guidelines were updated later on through expert consensus and became more specific and detailed regarding the terms under which surgeries could be postponed [1].

### Disease-centered & patient-oriented approach

Newly occurring challenges and subsequent alterations in healthcare services can provide clinicians an opportunity to reevaluate their approach to thyroid cancer management and encourage surgeons as well as patients to follow a more in-depth and data-driven decision-making process [30,31]. Admittedly, surgery for low-risk thyroid tumors can be safely delayed for a period of time while the patient remains under active surveillance or even offered an alternative, nonsurgical option [1,4,7,18,23,24,30]. However, it is important to note that a diagnosis of cancer upsets patients, let alone when asked to consider a conservative approach while their intuition is to undergo an immediate life-saving intervention [30]. Therefore, surgeons are tasked with helping patients gain a better understanding of their situation through a detailed and thorough consultation [18,30]. This includes an individualized risk–benefit analysis during which the surgeon can reassure the patient of the low-risk nature of the diagnosis and the efficacy of the suggested measure by referring them to specific trials and available online sources of information regarding their disease. Moreover, patients should be notified beforehand to be prepared and jot down questions they need clarified [18]. If extended observation is required, follow-up with ultrasound imaging can make patients more receptive to the idea of active surveillance [1,4].

### Anatomical concerns

Mild airway compression from large goiters is usually subacute or chronic and does not cause symptoms [32]. But what if a patient with a large goiter requires immediate intubation due to acute COVID-19-induced respiratory distress? Such a patient may present with significant and likely fatal airway compromise. Moreover, many patients with large goiters are extremely worried about their postponed surgeries not only for oncological reasons but for anatomical reasons as well.

Most differentiated and medullary thyroid cancers are slow-growing tumors that do not cause significant morbidity over the short-term, although there is a subset of more biologically aggressive cancers that progress more rapidly. Therefore, clinical correlation with rate of progression, size, invasion of surrounding structures and proximity to critical structures is recommended [33]. This means that for those living with thyroid malignancies – which are usually asymptomatic – the disease might continue to grow in the absence of treatment, and therefore the cancer may spread, lead to the need for more advanced operations and increase complication rates or, in some instances, lead to metastatic disease or even death [19,34,35]. Subsequently, the anatomical parameters play a vital role with regard to the decision for undelayed diagnosis and treatment.

## Conclusion

COVID-19 has caused unprecedented societal turmoil, triggering a rapid and still ongoing transformation of healthcare provision on a global level. In this new landscape, it is highly important to acknowledge the challenges this pandemic poses to the care of particularly vulnerable cancer patients and the subsequent psychosocial impact this has on them. Both diagnostic and therapeutic surgical procedures for thyroid cancer have declined during the COVID-19 pandemic. Even though active surveillance for a predefined time period can be a safe option for lower-risk patients, anaplastic, poorly differentiated, medullary and advanced differentiated thyroid cancer management must not be delayed.

## Future perspective

Prevention, timely diagnostic testing and effective treatment are key for the confinement of COVID-19 [6]. Notwithstanding the high importance of vaccinations, newly arising, more contagious and possibly more difficult to treat variants of the virus could pose an obstacle to the recovery from this pandemic, the duration of which is hard to predict [6,11,36]. Thus, thyroid cancer surgery guidelines need to be continuously updated based on the current phase of the pandemic while ensuring optimal safety for both patients and healthcare workers [11,16]. Hopefully, our experience with this modern-day pandemic will prove to be a valuable lesson for future crisis management as well as for everyday medicine.

**Executive summary**

**Effects on diagnosis**

- Diagnostic examinations have declined during the pandemic. The clinical picture should be taken into account when deciding to delay procedures.

**Recommendations for surgical triage**

- Most well-differentiated cancers can safely remain under active surveillance during acute pandemic phases. For more severe cases, surgery should still be an option. Alternative therapies can benefit carefully selected patients.

**Preoperative management**

- Coronavirus disease 2019 status screening and personal protective equipment can help protect both patients and personnel.

**Impact on surgical services**

- Thyroid cancer surgeries have decreased. Urgent oncology procedures are not impeded.

**Impact on follow-up**

- Coronavirus disease 2019 transmission has not been an issue. Telemedicine is a safe option for long-term follow-up.

**Protocol variation**

- Local discrepancies can be attributed to differences in coronavirus disease 2019 burden and hospital capacity. Guidelines were first based on previous experience and continue to be updated.

**Disease-centered & patient-oriented approach**

- An initial conservative approach is often a safe alternative to immediate surgery. Clinicians should explain to patients that their illness is managed after an in-depth risk–benefit analysis.

**Anatomical concerns**

- Complications can occur when diagnosis and treatment are delayed in the presence of pressure symptoms and local aggression.
